# Prevalence and Co-Occurrence of College Sexual Violence Experienced by 2SLGBTQIA+ Students: Gender- and Gender Modality-Based Analysis

**DOI:** 10.1177/08862605251368819

**Published:** 2025-10-09

**Authors:** Manon Bergeron, Marie-France Goyer, Alexa Martin-Storey, Matthieu Carignan-Allard, Geneviève Paquette, Martin Blais, Dominique Dubuc, Éric Kirouac, Geneviève Pagé, and the Alliance 2SLGBTQIA+ Project Team

**Affiliations:** 1 Université du Québec à Montréal, Canada; 2 Université de Sherbrooke, QC, Canada; 3 Cégep de Sherbrooke, QC, Canada; 4 Cégep Garneau, QC, Canada

**Keywords:** sexual violence, gender minority, sexual minority, college campus

## Abstract

Sexual and gender minorities are overrepresented among victims of sexual violence in higher education settings. This vulnerability encapsulates considerable variability, such that further information in required to tailor prevention and intervention approaches for those most vulnerable. The purpose of this study was to explore whether sexual violence experiences in college settings (SVCS) reported by sexual and gender minority students varied by gender identity (i.e., non-binary, women, men) and modality (i.e., trans or cisgender) of the participants. A total of 3,182 2SLGBTQIA+ participants studying in college settings in Quebec met the inclusion criteria and were included in the analyses. 54.1% of participants reported experiencing at least one SVCS since their arrival at the institution. Overall, cisgender sexual minority men were less likely to report experiencing at least one SVCS situation (vs. trans men, non-binary individuals, cisgender sexual minority women, gender-questioning individuals). Non-binary individuals reported higher co-occurrence of SVCS than cisgender sexual minority men and women. Compared to cisgender sexual minority men, gender-questioning individuals and cisgender sexual minority women reported higher co-occurrence of SVCS. Specific subgroup differences were also observed across gender identity and modality in terms of type of sexual violence reported. The findings support the need for SVCS prevention strategies that address the overlap between sexual violence and homophobic and transphobic discrimination, deconstruct heterocissexist norms, and specifically address the types of sexual violence most prevalent among 2SLGBTQIA+ victims (i.e., being stared at or sexually objectified; being told sexual stories or jokes).

Sexual violence in higher education settings affects a large number of students. In Canada, as elsewhere, research consistently suggests that sexual and gender minorities report higher levels of sexual violence than their heterosexual cisgender peers (Bergeron et al., 2016; Burczycka, 2020; Conroy & Cotter, 2017; Fedina et al., 2018; Lebugle et al., 2020; Linder et al., 2020). Several theories help to explain why sexual and gender minority individuals are more likely to experience sexual violence. First, minority stress theory, developed by Meyer (2003) and adapted to gender minorities by Hendricks and Testa (2012), propose that greater vulnerability to victimization among sexual and gender minorities results from the stigma associated with these identities. To this end, several researchers suggest that some acts of sexual violence are based on prejudices against sexual and gender minority individuals (Katz-Wise et al., 2013; Martin-Storey et al., 2022; Messinger & Koon-Magnin, 2019; Paquette, Castonguay-Khounsombath, et al., 2021; Richardson et al., 2015) and that 2SLGBTQIAphobic hate crimes often take the form of sexual violence (Ford & Soto-Marquez, 2016; Hutton, 2016; Pérez & Hussey, 2014). The way in which some 2SLGBTQIA+ (i.e., Two Spirit, Lesbian, Gay, Bisexual, Transgender, Questioning, Intersex, and Asexual+) people describe the sexual violence they experienced in higher education settings demonstrates an overlap between heterosexism, cissexism, and sexual violence (Martin-Storey et al., 2022).

Aligning with some feminist theories (Brownmiller, 1993; Donat & D’Emilio, 1992), sexual violence can function to reinforce gender-based hierarchies and can serve to punish and intimidate those who violate social norms (McPhail, 2016). Berdahl (2007) also suggests that sexual harassment functions to establish and maintain power hierarchies with respect to sex, gender presentation, and sexual behavior. Individuals with more power are more likely to commit sexual violence, while those with less power are more likely to be victimized (Martin-Storey et al., 2022).

Some studies suggest that among 2SLGBTQIA+ communities, certain groups are at greater risk for sexual violence, such as bisexual individuals (Burczycka, 2020; Coulter et al., 2017), particularly bisexual women (Brubaker et al., 2017; Cantor et al., 2020; Martin-Storey et al., 2018) and trans or non-binary individuals (Burczycka, 2020; Cantor et al., 2020; Coulter et al., 2017; Johnson et al., 2016; Martin-Storey et al., 2018; Richardson et al., 2015). Some theories help explain why trans or non-binary individuals are particularly vulnerable to sexual violence. Norm-centered stigma theory posits that the stigma experienced by trans or non-binary individuals is a result of privileging the gender binary, such that individuals outside of this binary are perceived as being on the margins of social acceptability (Worthen, 2021). Unfortunately, studies of sexual violence do not distinguish between the experiences of binary trans people (trans men and women) and non-binary people, with these groups systematically being merged in analyses (e.g. Burczycka, 2020; Cantor et al., 2020; Martin-Storey et al., 2018).

Sexual violence has consequences in many areas of the lives of victimized individuals, such as academic success, personal and social life, and physical and mental health (Burczycka, 2020; Dworkin, 2020; Théorêt et al., 2022). In this regard, sexual and gender minority students report more negative consequences such as depressive and trauma symptoms following situations of sexual violence than heterosexual cisgender students (Kammer-Kerwick et al., 2019; Paquette, Martin-Storey, et al., 2021; Smidt et al., 2021).

And, while sexual violence is a serious problem across the lifecourse, there are several reasons for focusing on sexual violence experienced by 2SLGBTQIA+ individuals in early adulthood. Young people aged 15 to 24 (Conroy & Cotter, 2017) and 2SLGBTQIA+ individuals (Burczycka, 2020; Conroy & Cotter, 2017; Fedina et al., 2018; Lebugle et al., 2020; Linder et al., 2020) are among the groups most at risk for sexual violence. The college years appear to be particularly ripe for sexual violence experienced by 2SLGBTQIA+ individuals.^1^ In order to promote appropriate prevention strategies, a better understanding of sexual violence experienced by sexual and gender minorities in the college setting is necessary.

This study uses an inclusive definition of sexual violence that includes different types of sexual violence, such as sexual (cyber)harassment, voyeurism or exhibitionism, dissemination of sexual images without consent, unwanted touching, sexual assault or threat of sexual assault, promises of rewards in exchange for sexual favors, and various forms of unwanted or non-consensual sexual behavior. This inclusive definition of sexual violence mirrors that advocated by the Government of Canada in the *National Framework to Prevent and Address Gender-Based Violence at Post-Secondary Institutions* (Khan et al., 2019), that was adopted by the Government of Quebec in 2017 in the *Act to Prevent and Fight Sexual Violence in Higher Education Institutions* (L22.1) (LQ, 2017). It is also consistent with the definition used by the World Health Organization (Krug et al., 2002) and the definition widely disseminated in the United States by the Centers for Disease Control and Prevention (Basile et al., 2014).

## Purpose of the Study

The literature suggesting general vulnerability for sexual violence experiences in college settings (SVCS) among sexual and gender minority college students contains numerous gaps. These include collapsing individuals with different gender minorities and/or gender minority identities together (Burczycka, 2020; Cantor et al., 2020; Edwards et al., 2015; Martin-Storey et al., 2018) and focusing on a narrow range of types of sexual violence (Coulter & Rankin, 2020; Eisenberg et al., 2021). Using a large sample of 2SLGBTQIA+ college students and a detailed measure of multiple forms of sexual violence, the purpose of this study is to explore whether prevalence, co-occurrence, and types of SVCS experienced by sexual and gender minority students vary by gender identity and modality. In this study, the term “college settings” captures actions committed on or off campus against a student by an individual affiliated with the same institution.

## Methods

The objective of the current study was to provide a better understanding of the SVCS experienced by individuals in the 2SLGBTQIA+ communities by exploring the different types of SVCS by gender identity (i.e., non-binary, woman or man) and modality (trans or cisgender).^2^ Data were obtained from the *Alliance 2SLGBTQIA+ : pour une culture de respect, d’égalité et de consentement en milieu collégial* project (Bergeron et al., 2023). The research project received approval from the Comité institutionnel d’éthique de la recherche avec des êtres humains of the Université du Québec à Montréal (No. 4909_e_2021) and from 32 additional ethics committees associated with 42 college institutions.

Participants were invited to complete an online survey assessing sexual violence among students attending general vocational colleges in Quebec, Canada. These Quebec-specific postsecondary institutions primarily offer 2-year preuniversity programs or 3-year technical diplomas. With some exceptions, a diploma from one of these institutions is required for Quebec students entering university. The two inclusion criteria for completing the online questionnaire were that the participant was studying at a Quebec college institution at the time of data collection and that the participant identified as a 2SLGBTQIA+ person. Therefore, only non-cisgender, non-exclusively heterosexual, or intersex individuals were invited to continue with the questionnaire beyond the sociodemographic section.

Participants were recruited between October of 2021 and May of 2022. Different recruitment strategies were employed: emailing through institutional mailing lists and invitations published on the intranet platform of Quebec colleges (more than 50 colleges distributed the call for participation to the entire student community), emailing through community organization mailing lists, sharing on social networks, etc. In addition, an incentive was offered to encourage participation. Forty $25 CAD (Interac transfer) prizes were offered. At the end of the questionnaire, individuals wishing to participate in the draw could click on a link to another form not linked to the study database to provide their name and contact information.

A total of 7,395 accesses to the survey were logged, of whom 5,444 indicated that they were eligible to participate due to the two selection criteria. Of these remaining individuals, a total of 2,241 were excluded because of two reasons. First, due to the objectives of the survey, we excluded questionnaires for which the mandatory questions of the first two sections (sociodemographic data and SVCS) were not completed (*n* = 2,100). Then, we removed 89 duplicates, 34 questionnaires with non-serious or hateful text responses, 2 questionnaires with improbable quantitative response patterns (e.g., 1, 2, 3, 4, 5; 1, 2, 3, 4, 5, etc. or 5, 5, 5, 5, 5, etc.), and 16 questionnaires completed by participants who did not meet the inclusion criterion, i.e., a person from a sexual or gender minority. For these reasons, the overall sample for the *Alliance 2SLGBTQIA+* project consists of 3,203 2SLGBTQIA+ individuals studying in college settings. Additional methodological details can be found in the research report (Bergeron et al., 2023) available on https://chairevssmes.uqam.ca/. In line with study objectives, only participants who answered the sociodemographic questions allowing us to identify their gender identity and modality were included in the sample of the present article, for a total of 3,182 participants.

### Measures

#### Sexual Victimization in the College Setting

To document the frequency of SVCS, we used the French version of the Sexual Experiences Questionnaire (Fitzgerald et al., 1999), translated by the ESSIMU team (Bergeron et al., 2016). This validated instrument distinguishes between three categories of sexual violence: (a) sexual harassment (verbal and non-verbal behaviors that are not aimed at sexual cooperation, but result in insulting, hostile, and degrading attitudes); (b) unwanted sexual behaviors (offensive, unwanted, and non-reciprocal verbal and non-verbal behaviors that include attempted rape and sexual assault); and (c) sexual coercion (being pressured, tricked, threatened, or forced to engage in sexual activity in return for future employment or educational considerations).

To ensure that the measure was tailored to 2SLGBTQIA+ individuals, the research team reviewed the results of the analysis of 2SLGBTQIA+ sexual violence narratives from the ESSIMU survey (Paquette, Castonguay-Khounsombath, et al., 2021) and consulted with four community organizations with expertise in the field (Diversité 02, RÉZO, CQ-LGBT, Interligne – Lexic^2^ project). These initiatives led to two main adjustments: The addition of a novel item on sexual harassment germane to sexual and gender minority populations (“Asked you intrusive or persistent questions about your sexuality or body”) and the addition of a item on sexual coercion germane to sexual and gender minority populations (“Revealed or threatened to reveal your sexual orientation, trans background, or any other intimate information [e.g., sexual preferences, HIV status] if you refused to engage in sexual activities”).

The final measure combines 22 items (Cronbach’s alpha coefficient (α) = .87; confidence interval (CI) [0.86, 0.88]) distributed as follows: 9 items for sexual harassment (α = .80; CI [0.79, 0.81]), 7 for unwanted sexual behavior (α = .80; CI [0.79, 0.81]), and 6 for sexual coercion (α = .82; CI[0.81, 0.83]). For each statement, the individual was asked to indicate whether someone affiliated with the same college had committed this act toward them, since their arrival at the institution. The response options were “never”, “once” and “twice or more”. For prevalence analyses, responses were recoded dichotomously (yes/no). A co-occurrence score was also created. Co-occurrence refers to the number of different SVCS statements selected by the participants (score 1–22). Co-occurrence was analyzed only among individuals who experienced at least one SVCS event.

#### Gender Identity and Modality

First, participants chose the term(s) that best defined their gender identity from a list of 10 options: agender, bigender, gender creative, woman or girl, gender fluid, man or boy, non-binary, queer, Indigenous or culturally specific gender identity, and uncertain/questioning. Participants could select more than one response option or add another response. Gender modality (cisgender or trans) was then measured using the question, “Do you identify with the gender you were assigned at birth?” The response options were: yes, no, unsure, or questioning. Gender identity and gender modality were cross-tabulated to create a variable distinguishing the following six categories: (a) trans woman, (b) cisgender woman, (c) trans man, (d) cisgender man, (e) non-binary individual (includes those who selected the following response options: non-binary, queer, gender fluid, agender, gender creative, bigender, culturally specific and/or other gender identity, or selected both man and woman), and (f) unsure/questioning.

#### Sexual Orientation

Participants chose the term(s) that best defined their sexual orientation from a list of 12 options: allosexual; asexual; bicurious; bisexual; gay, lesbian or homosexual; heteroflexible; heterosexual; homoflexible; pansexual; queer; Indigenous or culturally specific sexual orientation; and unsure/questioning. Participants could select more than one sexual orientation option or add another response. Responses were recoded into four categories: (a) gay, lesbian, or homosexual (exclusively), (b) heterosexual (exclusively), (c) not exclusively gay, lesbian or heterosexual, and (d) uncertain/questioning. Although no analysis by sexual orientation is presented in this article, it is important to remember that the sample is composed solely of 2SLGBTQIA+ individuals. Thus, the cisgender individuals in the sample were not exclusively heterosexual and the exclusively heterosexual individuals were gender minorities.

#### Other Sociodemographic Characteristics

The following sociodemographic characteristics were also documented: age, duration of attendance at the institution, student status, migration status, belonging to a racialized community, identification as Indigenous, and medically confirmed intersex variation. These variables were used to describe the sociodemographic profile of the entire sample (Table 1).

**Table 1.** Frequency of Sociodemographic Characteristics of the Quantitative Sample (*n* = 3,182).

Variable	*n*	%
Age	**3,175**	
17 years old and under	724	22.8
18 to 25 years old	2,201	69.3
26 to 35 years old	200	6.3
36 to 45 years old	43	1.4
46 to 55 years old	7	0.2
Duration of attendance at the institution	**3,182**	
1 to 2 sessions	1,498	47.1
3 to 4 sessions	1,008	31.7
5 to 6 sessions	488	15.3
7 to 8 sessions	132	4.1
9 sessions or more	56	1.8
Gender identity and modality	**3,182**	
Trans woman	39	1.2
Cisgender woman	1,586	49.8
Trans man	91	2.9
Cisgender man	468	14.7
Non-binary individual	547	17.2
Uncertain/questioning	451	14.2
Intersex^a^	**3,163**	
No	3,149	99.6
Yes	14	0.4
Sexual orientation	**3,166**	
Gay, lesbian, or homosexual (exclusively)	544	17.2
Heterosexual (exclusively)	37	1.2
Not exclusively gay, lesbian, or heterosexual^b^	2,322	73.3
Uncertain/questioning	263	8.3
Student status	**3,175**	
Regular	3,023	95.2
International	152	4.8
Migration status	**3,178**	
Born in Canada	2,767	87.1
Born outside of Canada	407	12.8
Uncertain	4	0.1
Belonging to a racialized community	**3,169**	
Yes	326	10.3
No	2,741	86.5
Uncertain	102	3.2
Identification as Indigenous	**3,123**	
Yes	87	2.8
No	3,036	97.2

*Note.*

a Due to the small sample of intersex people, the analyzes do not take into account intersex status.

b The category “Not exclusively gay, lesbian or heterosexual” includes those who selected the following response options: asexual or on the asexual spectrum, bicurious, bisexual, heteroflexible, homoflexible, pansexual, queer, Indigenous or culturally specific sexual orientation (e.g., two-spirited), and/or other sexual orientation.

### Participant Profile

Table 1 presents the sociodemographic characteristics of the sample. The majority of students in the sample are not exclusively gay, lesbian, or heterosexual, meaning that they are attracted to more than one gender or fall on the asexual spectrum (73.3 %). The majority of the sample is cisgender (64.5%), and half of the sample is between the ages of 18 and 25 and has been attending college for one to four semesters.

### Analyses

Bivariate analyses were conducted to test whether SVCS experiences differed based on participants’ gender identity and modality. For categorical variables (e.g., presence/absence of SVCS), Chi-square tests were conducted. The strength of associations was estimated using Cramér’s *V* (≤ 0.10 *V* = *negligible association*; 0.10 ≤ *V* ≤ 0.20 = *weak association*; ≤ 0.20 *V* ≤ 0.40 = *moderate association*; 0.40 ≤ *V* ≤ 0.60 = *relatively strong association*; 0.60 ≤ *V* ≤ 0.80 = *strong association*; 0.80 ≤ *V* ≤ 1.00 = *very strong association*; Rea & Parker, 1992). If an association was detected as statistically significant, multiple comparison (pairwise) tests were performed to detect the group(s) that differed significantly from others. When cell sizes were too small to conduct Chi-square tests, Fisher exact tests were performed. For continuous variables (co-occurrence of SVCS), non-parametric Kruskal–Wallis tests (which do not assume normality of data distributions) were used. The significance level was set at 0.05 for all tests. Analyses were conducted on IBM SPSS Statistics software, version 29.

## Results

### Prevalence of SVCS

A total of 54.1% of participants reported at least one experience of sexual violence, with sexual harassment being the most reported form of SVCS. Table 2 presents the percentage of participants who reported experiencing sexual violence (sexual harassment, unwanted sexual behavior, sexual coercion, and overall experiences of SVCS) for the overall sample and by gender identity and modality. Overall, cisgender sexual minority men were less likely than other groups to report at least one situation of SVCS (*p* < .001). Specifically trans men and non-binary individuals were more likely to have reported *sexual harassment* than cisgender sexual minority women and cisgender sexual minority men (*p* < .001). Non-binary individuals, cisgender sexual minority women, and gender-questioning individuals were more likely to have reported *unwanted sexual behavior* than cisgender sexual minority men (*p* < .001). Finally, non-binary individuals were more likely to have reported *sexual coercion* than cisgender sexual minority men (*p* = .047). No other signification differences were observed. For all observed differences, the effect size was from negligible (unwanted sexual behavior and sexual coercion scores) to weak (sexual harassment and overall sexual violence scores).

**Table 2.** Distribution of Types of Sexual Violence in a College Setting Reported Since Arrival at the Institution, Based on Gender Identity and Modality (*n* = 3,182). .

	Total *n* = 3,182	Cis W *n* = 1,586	Cis M *n* = 468	Trans W *n* = 39	Trans M *n* = 91	NB *n* = 547	Quest. *n* = 451	Statistic	
Type of SVCS	%	%	%	%	%	%	%	*p*	*V*
**Sexual harassment**	**51.4**	51.1_a_	40.6_b_	48.7_a,b,c,d_	69.2_d_	58.7_c,d_	51.0_a,c_	<0.001	0.12
Stared at you or sexually objectified you in a way that made you uncomfortable.	**27.0**	29.5_a_	13.7_b_	28.2_a,b_	31.9_a_	30.0_a_	27.1_a_	<0.001	0.13
Repeatedly told you sexual stories or jokes that offended or embarrassed you.	**23.6**	22.2_a,b_	17.7_b_	25.6_a,b,c_	23.1_a,b,c_	30.5_c_	26.4_a,c_	<0.001	0.09
Made derogatory remarks to you about your appearance, body, or actual or suspected sexual activity.	**19.7**	19.0_a,b_	13.7_b_	17.9_a,b,c_	30.8_a,c_	25.0_c_	20.0_a,b,c_	<0.001	0.09
Tried to start a discussion about sex with you, even if you didn't like it (e.g., tried to discuss your sex life with you).	**18.6**	17.3_a_	15.2_a_	10.3_a,b_	25.3_a,b_	23.2_b_	20.8_a,b_	0.002	0.08
Asked you intrusive or persistent questions about your sexuality or body.	**17.2**	14.9_a_	14.5_a_	23.1_a,b,c_	33.0_c_	22.3_b,c_	18.2_a,b_	<0.001	0.11
Made insulting or hurtful sexual comments to you.	**14.3**	13.3_a_	10.0_a_	10.3_a,b_	14.3_a,b_	19.6_b_	16.2_a,b_	<0.001	0.08
Whistled at you or called you names in a sexual way.	**10.2**	11.6_a,b_	4.3_c_	2.6_a,b,c_	2.2_b,c_	13.3_a_	10.0_a,b_	<0.001	0.11
Made sexually suggestive gestures that offended or embarrassed you.	**7.5**	8.1_a_	3.6_b_	7.7_a,b_	2.2_a,b_	9.0_a_	8.6_a_	0.004	0.07
Exposed themself naked or scantily clad in a way that made you feel uncomfortable.	**3.4**	3.2	4.1	2.6	5.5	4.4	2.2	0.335	0.04
**Unwanted sexual behavior**	**20.0**	21.6_a_	12.4_b_	10.3_a,b_	13.2_a,b_	23.0_a_	20.8_a_	<0.001	0.09
Touched you in a way that made you feel uncomfortable.	**10.4**	10.5_a_	4.5_b_	5.1_a,b_	5.5_a,b_	13.9_a_	13.3_a_	<0.001	0.10
Sent you by Internet (or other technology) or exposed you to sexually suggestive material (pictures, writing, objects, videos) to which you did not want to be exposed.	**7.4**	8.1	4.5	2.6	6.6	9.0	6.9	0.062	0.06
Repeatedly asked you out for drinks or to eat, despite your refusal.	**7.4**	8.8_a_	2.6_b_	2.6_a,b,c_	0.0_b,c_	9.1_a_	7.8_a,c_	<0.001	0.10
Tried to establish an intimate or sexual relationship with you, despite your refusal or efforts to discourage it.	**7.2**	8.0_a_	3.2_b_	2.6_a,b_	3.3_a,b_	9.5_a_	7.1_a,b_	0.001	0.08
Tried to touch, kiss, or rub against you when you did not want them to.	**7.0**	7.7_a_	2.6_b_	2.6_a,b_	3.3_a,b_	8.2_a_	8.9_a_	<0.001	0.08
Tried to have sex with you when you did not want to, but failed.	**3.3**	3.7	1.9	0.0	1.1	3.3	4.0	0.217	0.05
Engaged in sexual activity with you when you did not want to.^a^	**1.9**	1.8	0.6	0.0	3.3	2.9	2.0	0.084	0.05
**Sexual coercion**	**4.7**	4.9_a,b_	2.4_b_	0.0_a,b_	3.3_a,b_	6.2_a_	5.1_a,b_	0.047	0.06
Suggested that you would be better treated if you cooperated sexually.^a^	**2.0**	2.0_a,b_	0.6_b_	0.0_a,b_	0.0_a,b_	3.3_a_	2.4_a,b_	0.036	0.06
Suggested that you would be rewarded for a future exchange of sexual favors.^a^	**1.6**	1.7	0.4	0.0	2.2	2.7	1.1	0.059	0.06
Made you suffer negative consequences for refusing to engage in sexual activity.^a^	**1.6**	1.7	0.4	0.0	1.1	2.2	2.0	0.204	0.05
Made you fear retaliation if you refused to engage in sexual activity.^a^	**1.5**	1.4	0.4	0.0	0.0	2.2	2.4	0.075	0.06
Revealed or threatened to reveal your sexual orientation, trans background, or other intimate information (e.g., sexual preferences, HIV status) if you refused to engage in sexual activity.^a^	**1.3**	1.1	1.1	0.0	1.1	2.2	1.3	0.491	0.04
Made sure that you were afraid of the consequences if you refused to engage in sexual activity.^a^	**1.2**	1.1_a,b_	0.0_b_	0.0_a,b_	0.0_a,b_	2.0_a_	1.8_a,b_	0.019	0.06
**At least one form of SVCS**	**54.1**	54.6_a_	43.4_b_	48.7_a,b_	69.2_a_	59.8_a_	53.7_a_	<0.001	0.11

*Note.* The *V* symbol is used to represent Cramér’s V.

When two groups do not share the same index letter, it indicates that there is a statistically significant difference between these groups (*p* < .05). The comparison of letters should be performed horizontally.

a Due to small theoretical numbers, Fisher tests were performed for these variables.

### Types of SVCS

Table 2 also presents the percentage of participants who reported each type of SVCS for the overall sample and by gender identity and modality. The most common types of SVCS reported by participants were “Stared at you or sexually objectified you in a way that made you uncomfortable”, “Repeatedly told you sexual stories or jokes that made you feel offended or embarrassed”, “Made derogatory remarks to you about your appearance, body, or actual or suspected sexual activity”, “Tried to start a discussion about sex with you, even if you didn’t like it (e.g., tried to discuss your sex life with you)”, and “Asked you intrusive or persistent questions about your sexuality or body.” Some types of SVCS were reported in higher proportions by some groups. Trans men, non-binary individuals, cisgender sexual minority women, and gender-questioning individuals were more likely to report being stared at or sexually objectified in a way that made them feel uncomfortable than cisgender sexual minority men (*p* < .001). Also, non-binary individuals were more likely to have been repeatedly told sexual stories or jokes that made them feel offended or embarrassed than cisgender sexual minority women and cisgender sexual minority men (*p* < .001). Trans men and non-binary individuals were more likely to report hearing derogatory remarks about their actual or perceived appearance, body, or sexual activity than cisgender sexual minority men (*p* < .001). They were also more likely to have report hearing intrusive or persistent questions about their sexuality or body than cisgender sexual minority women and cisgender sexual minority men (*p* < .001). The effect size for all observed differences was weak or negligible.

While the proportion of individuals reporting at least one instance of SVCS was similar between groups (with the exception of cisgender sexual minority men who experienced less), some groups were exposed to a greater diversity of acts of SVCS (co-occurrence).

### Co-Occurrence of SVCS

The co-occurrence of SVCS was measured among individuals reporting at least one incidence of SVCS (Table 3). Co-occurrence was compared across participants’ gender identity and modality (Table 4). The results reveal a significant difference according to participants’ gender identity and modality (*X*^2^_(5)_ = 17.34; *p* = .004). Specifically, the results of the multiple (pairwise) comparisons show that compared to cisgender sexual minority men (Mean (*M*) = 2.76; Standard Deviation (*SD*) = 2.09; Median (*MD*) = 2.00), non-binary individuals (*M* = 4.15; *SD* = 3.80; *MD* = 3. 00) (*p* < .001), gender-questioning individuals (*M* = 3.92; *SD* = 3.63; *MD* = 2.00) (*p* = .004), and cisgender sexual minority women (*M* = 3.60; *SD* = 3.24; *MD* = 2.00) (*p* = .006) reported more co-occurrence of SVCS. Non-binary individuals also reported more co-occurrence of SVCS than cisgender sexual minority women (*p* = .034). No other significant differences were observed between these groups.

**Table 3.** Co-Occurrence of Acts of SVCS Experienced Since Arrival at the Institution, by Gender Identity and Modality (*n* = 1,720).

	Number of different acts of SVCS experienced		
Gender identity and modality	Mean (*M*)	Standard deviation (*SD*)	Median (*MD*)
Non-binary (*n* = 327)	4.15	3.80	3.0
Questioning (*n* = 242)	3.92	3.63	2.0
Cis woman (*n* = 866)	3.60	3.24	2.0
Trans woman (*n* = 19)	2.95	2.01	2.0
Trans man (*n* = 63)	2.83	2.00	2.0
Cis man (*n* = 203)	2.76	2.09	2.0

**Table 4.** Multiple (Pairwise) Comparisons of Co-Occurrence of Acts of SVCS Experienced Since Arrival at the Institution, by Gender Identity and Modality (*n* = 1,720).

Gender identity and modality	Cis woman (*n* = 866)	Cis man (*n* = 203)	Trans woman (*n* = 19)	Trans man (*n* = 63)	Non-binary (*n* = 327)	Questioning (*n* = 242)
	*p*	*p*	*p*	*p*	*p*	*p*
Cis woman (*n* = 866)	-	0.006	0.846	0.344	0.034	0.445
Cis man (*n* = 203)	-	-	0.477	0.522	<0.001	0.004
Trans woman (*n* = 19)	-	-	-	0.764	0.440	0.673
Trans man (*n* = 63)	-	-	-	-	0.058	0.206
Non-binary (*n* = 327)	-	-	-	-	-	0.335
Questioning (*n* = 242)	-	-	-	-	-	-

## Discussion

In line with the objective of the current study, findings indicated that prevalence and co-occurrence of SVCS varied by gender identity and modality and that non-binary people constitute a particularly vulnerable group for SVCS. Non-binary people were among the most likely to have reported at least one incident of sexual harassment and unwanted sexual behavior (prevalence). They had also been exposed to more types of SVCS (co-occurrence) than sexual minority cisgender women and men. These results are consistent with the work of Worthen (2020, 2021), which demonstrate that non-binary people endure unique stigmatization related to their non-normative identities and their existence beyond the gender binary system.

Results showed that sexual harassment is the most common category of SVCS experienced by the 2SLGBTQIA individual in this study. More specifically, the most frequent SVCS experiences included being stared at or sexually objectified, being told sexual stories or jokes repeatedly, hearing derogatory remarks about their appearance, body, or real or perceived sexual activity; being forced to discuss sex; and being asked intrusive or persistent questions about their sexuality or body. These experiences are similar to those identified by Martin-Storey et al. (2022) in the analysis of qualitative stories from the ESSIMU survey on sexual violence in a university setting (e.g. jokes, intrusive questions). Some of these experiences of sexual harassment (e.g., staring and derogatory remarks about appearance, body, or sexual activities) suggest an overlap between sexual harassment and 2SLGBTQIA-phobias.

Although differences between groups were negligible or weak, some types of SVCS differences in specific experiences were observed across groups. For example, trans men and non-binary individuals were particularly likely to have been stared at or sexually objectified in a way that made them feel uncomfortable, to have heard derogatory remarks about their appearance, body, or real or perceived sexual activity, or to have experienced intrusive or persistent questions about their sexuality or body. In accordance with the feminist theories presented in the introduction (McPhail, 2016), the nature of this variation in vulnerability suggests that trans or non-binary individuals are targets of sexual violence because they transgress gender norms. Sexual harassment, then, may reflect a way of policing conformity to norms by individuals in these groups, by establishing and maintaining power hierarchies regarding sex, gender presentation, and sexual behavior (Berdahl, 2007).

### Contributions and Limitations of the Study

This study has several methodological strengths. First, it deepened our understanding of SVCS experienced by 2SLGBTQIA+ individuals, a group overrepresented among victims of sexual violence in higher education settings. Access to a large sample of 2SLGBTIA+ individuals gave us the opportunity to explore variation in the experience of SVCS by gender identity and modality, allowing for the development of recommendations tailored for the needs of specific groups. Second, the measure used to document SVCS (in three dimensions: sexual harassment, unwanted sexual behavior, and sexual coercion) and its adaptation following consultation with 2SLGBTQ+ organizations allowed for the identification of a broad inventory of sexual violence experiences reported by 2SLGBTQIA+ individuals. However, certain methodological limitations must be considered. Since many individuals in gender and sexual minority communities are reluctant answering questions about sex assigned at birth, based on the strong recommendations of our advisory table consisting of community partners, we did not collect information about this variable. Likewise, we did not collect information about trans or non-binary participants' ability to pass, i.e., the ability to be perceived as cisgender according to sociocultural gender norms (Matsuzaka & Koch, 2019). According to a recent study by Matsuzaka and Koch (2019), the ability to pass influences the type of sexual victimization experienced by trans individuals. For example, trans women who have the privilege to pass report experiences of sexual violence similar to cisgender women, while those who do not pass report experiences of objectification, fetishization, devaluation, and transphobic aggression (Matsuzaka & Koch, 2019). And, while the sample employed included a large number of 2SLGBTQIA+ young people, the sample was largely non-racialized and non-Indigenous, which prevented exploring how these identities, as well as sexual and gender minority identities specific to those communities like Two Spirit identities are linked to variation in sexual violence. Finally, although the current study had a large sample, the participants were likely not representative of the entire 2SLGBTQIA+ population studying at a Quebec college.

### Implications

Findings from the current study suggest several practical recommendations. First, both the high prevalence and the greater variety of SVCS experienced by non-binary individuals suggest the vulnerability for this group in terms of their sense of safety on campus and remind us that additional efforts must be made (in terms of SVCS prevention, discrimination, and inclusion) to ensure the safety of this segment of the 2SLGBTQIA+ population specifically. The results also highlight the importance of institutions of higher education working with gender minority students to develop prevention policies and strategies that address their specific needs (Martin-Storey et al., 2018; Paquette, Martin-Storey, et al., 2021; Rothman et al., 2011). The campus-based sexual violence prevention literature is increasingly informed by the specific vulnerability of 2SLGBTQIA+ students (Bonar et al., 2022). Prevention programming, moreover, focus on whole school approaches that involve consultation with students, teachers, faculty, staff, institutional leadership, parents and significant adults, and community partnerships to develop programming appropriate to specific contexts (McMahon et al., 2020, 2021). This may be particularly the case for 2SGLBTQIA+ individuals, with research demonstrating how individuals from historically marginalized groups underscore how addressing this marginalization is essential for addressing vulnerability to campus-based sexual violence. To this end, prevention strategies (policies, programs, and interventions) and stakeholders responsible for accompanying victims of SVCS should consider the overlap between sexual violence (especially sexual harassment) and homophobic and transphobic discrimination (Martin-Storey et al., 2022). Strategies to prevent sexual violence should address transphobia and seek to deconstruct heterocissexist norms that marginalize trans or non-binary people should be privileged. Similarly, programs aimed at preventing transphobia should address sexual violence since sexual violence can be used to improve campus climate for these populations, and should be tested for the efficacy in addressing sexual violence specifically. As Martin-Storey et al. (2022) suggest, professionals working with 2SLGBTQIA+ individuals who have experienced SVCS need to consider the overlap between sexual violence and discrimination by exploring the meaning that victimized individuals attach to these experiences without attempting to classify these experiences for them (Martin-Storey et al., 2022). Finally, the results support the inclusion of content in SVCS awareness and prevention activities that specifically addresses the types of SVCS most frequently experienced by 2SLGBTQIA+ individuals (i.e., being stared at or sexually objectified, being told sexual stories or jokes, derogatory remarks about appearance, body, or sexual activity, etc.).

This recommendation would make it possible to adapt prevention strategies to the realities and needs of 2SLGBTQIA+ individuals and to inform the public about the intrusive nature of certain behaviors (inappropriate looks, jokes, intimate questions, etc.).

## Conclusion

By exploring the experiences of sexual violence in college settings (SVCS) reported by sexual and gender minority students and the variations by participants’ gender identity and modality, our study identified the most prevalent types of SVCS among sexual and gender minority individuals and the heightened vulnerability of non-binary students. These findings provide avenues for improving SVCS prevention and feelings of safety for 2SLGBTQIA+ individuals studying in college settings. We recommend that future research examines the role of gender expression, ability to pass, and transgression of binary gender norms on sexual violence experienced by trans and non-binary people.

## Notes

1. In Québec, college is the first level of education at which schooling is not compulsory. College studies follow secondary school and precede university education. They lead to obtaining a college studies diploma (pre-university program: 2 years full-time; technical program: 3 years full-time) or an attestation of college studies (short-term technical programs) (Government of Quebec, 2023).

2. Gender modality refers to the concordance (or lack thereof) between sex assigned at birth and gender identity. When these two dimensions agree, the gender modality is called cisgender; when they do not match, the gender modality is called transgender (Ashley, 2022; Blais et al., 2022).
